# Smart-Data-Glove-Based Gesture Recognition for Amphibious Communication

**DOI:** 10.3390/mi14112050

**Published:** 2023-10-31

**Authors:** Liufeng Fan, Zhan Zhang, Biao Zhu, Decheng Zuo, Xintong Yu, Yiwei Wang

**Affiliations:** 1 School of Computer Science and Technology, Harbin Institute of Technology, Harbin 150001, China; fanliufeng@ftcl.hit.edu.cn (L.F.); zhangzhan@hit.edu.cn (Z.Z.); yuxintong@ftcl.hit.edu.cn (X.Y.); 22s103226@stu.hit.edu.cn (Y.W.); 2 Department of Electronic and Information Science, University of Science and Technology of China, Hefei 230052, China; zhubiao7678@163.com

**Keywords:** hand gesture recognition, smart data glove, underwater gesture recognition, amphibious communication, deep learning, transfer learning

## Abstract

This study has designed and developed a smart data glove based on five-channel flexible capacitive stretch sensors and a six-axis inertial measurement unit (IMU) to recognize 25 static hand gestures and ten dynamic hand gestures for amphibious communication. The five-channel flexible capacitive sensors are fabricated on a glove to capture finger motion data in order to recognize static hand gestures and integrated with six-axis IMU data to recognize dynamic gestures. This study also proposes a novel amphibious hierarchical gesture recognition (AHGR) model. This model can adaptively switch between large complex and lightweight gesture recognition models based on environmental changes to ensure gesture recognition accuracy and effectiveness. The large complex model is based on the proposed SqueezeNet-BiLSTM algorithm, specially designed for the land environment, which will use all the sensory data captured from the smart data glove to recognize dynamic gestures, achieving a recognition accuracy of 98.21%. The lightweight stochastic singular value decomposition (SVD)-optimized spectral clustering gesture recognition algorithm for underwater environments that will perform direct inference on the glove-end side can reach an accuracy of 98.35%. This study also proposes a domain separation network (DSN)-based gesture recognition transfer model that ensures a 94% recognition accuracy for new users and new glove devices.

## 1. Introduction

With the continuous development of wearable sensor technology, human–computer interaction (HCI) has become an important research area in computer science. As an essential branch of HCI, gesture recognition technology can be applied to various fields, such as smart homes [1], intelligent driving [2], sign language recognition [3], virtual reality [4], and drone control [5]. With the continuous improvements in gesture recognition technology, this technology can also be used in amphibious environments to complete some tasks, such as communication with divers and underwater operations [6].

Although traditional vision-based gesture recognition technology has matured, it has significant limitations in underwater environments [7,8]. The cost of underwater cameras is high, the underwater shooting environment is complex, and it is very easy to be disturbed by water flow, water bubbles, etc., which hinder the line of sight and make shooting difficult. Sensor-based gesture recognition technology has become popular for underwater gesture recognition because of its lower cost and higher stability (not easily affected by the underwater environment). It has become a research area that many researchers are interested in. However, sensor-based gesture recognition technologies still face many challenges in amphibious environments.

First, the environment could affect the sensors, leading to some discrepancies between the gesture data collected on land and underwater. In the underwater environment, factors such as water pressure, water flow, and water quality will affect the sensors, affecting the accuracy and integrity of data collection. Secondly, people will feel more resistance and pressure due to the increased water depth, resulting in slow or non-standard gestures. Thirdly, there needs to be more accuracy when using a pretrained recognition model to perform cross-user and cross-device gesture recognition. Fourthly, Bluetooth signals cannot transmit underwater, and how to collect data and recognize real-time gestures underwater is also a problem that needs to be solved. Finally, although several existing data gloves are used for gesture recognition, none are used for amphibious environments. The above challenges must be considered when designing and selecting a gesture recognition model to ensure accuracy, robustness, and reliability.

This paper addresses the above research gaps by developing a smart data glove integrating environmental sensors, five-channel capacitive flexible stretch sensors, and six-axis IMU (three-axis accelerometer and three-axis gyroscope) and proposing a novel hierarchical hand gesture recognition model. The proposed model introduces a novel SqueezeNet-BiLSTM algorithm, a large complex recognition algorithm designed for land gesture recognition, and another lightweight stochastic SVD-optimized algorithm designed for underwater gesture recognition, which will be directly applied to the glove-end side. Additionally, this study introduces the DSN-based transfer learning for gesture recognition to ensure the recognition accuracy of new users and new glove devices. This paper makes the following contributions:

- A new smart data glove integrating environmental sensors, five-channel capacitive flexible stretch sensors, and six-axis IMU (three-axis accelerometer and three-axis gyroscope).
- A novel amphibious hierarchical gesture recognition (AHGR) model that can adaptively switch the classification algorithm based on the environment (underwater and land) between
  ○ a complex SqueezeNet-BiLSTM classification algorithm for land gesture recognition and
  ○ a lightweight stochastic SVD-optimized spectral clustering classification algorithm for underwater gesture recognition.
- A domain separation network (DSN)-based gesture recognition transfer model to ensure the recognition accuracy of new users and new glove devices.

The rest of the paper is organized as follows: Section 2 provides a review of related work. Section 3 introduces this study’s proposed smart data glove and predefined gesture set. Section 4 describes the proposed amphibious hierarchical gesture recognition model. Section 5 describes the proposed DSN-based gesture recognition transfer model. Section 6 presents the experimental results and analysis. Section 7 concludes this paper.

## 2. Related Work

### 2.1. Sensor-Based Gesture Recognition

Sensor-based gesture recognition can be roughly divided into the following four types: surface electromyography (sEMG) signal-based gesture recognition, IMU-based gesture recognition, stretch-sensor-based gesture recognition, and multi-sensor-based gesture recognition.

sEMG usually records the combined effect of the electromyographic signal of the surface muscle and the nerve trunk’s electrical activity on the skin’s surface. sEMG-based gesture recognition usually relies on surface electrodes deployed on the human arm or forearm to collect sensor signals [9,10,11,12]. However, sEMG-based gesture recognition also has some drawbacks. Firstly, the signals correlate strongly with the user’s status, leading to unstable recognition results. Secondly, the collection of sEMG signals requires the electrodes to be tightly attached to the user’s skin, and prolonged use is susceptible to the influence of oils and sweat produced by the user’s skin and makes users uncomfortable.

IMU-based gesture recognition mainly uses one or more combinations of accelerometers, gyroscopes, and magnetometers to collect hand movement information in the space field [13]. Siddiqui and Chan [14] used the minimum redundancy and maximum correlation algorithm to study the optimal deployment area of the sensor, deployed the sensor on the user’s wrist, and proposed a multimodal framework to solve the IMU sensing during the gesture movement bottleneck problem. Galka et al. [15] placed seven inertial sensors on the experimenter’s upper arm, wrist, and finger joints, proposed and used a parallel HMM model, and reached a recognition accuracy of 99.75%. However, inertial sensors still have limitations, and they focus more on spatial dimension information, which is mainly used for coarse-grained gesture recognition of large gesture movements. It is challenging to perform finer-grained segmentation and recognition, such as recognition of the degree of bending of finger joints.

Flexible stretch-sensor-based gesture recognition is usually used to record changes in gesturing finger joints. Stretch sensors are often highly flexible, thinner, and more portable than other sensors [16,17]. Therefore, in recent years, research on gesture recognition technology based on stretch sensors has also received extensive attention from researchers. However, the limitations of flexible stretch sensors are also evident. First, they can only capture hand joint information but cannot capture the spatial motion characteristics of gestures. Second, stretch sensors are usually sensitive, so they are more prone to damage, and the data they generate are more prone to bias than those from other sensors.

Although the above three sensor-based gesture recognition methods can achieve remarkable gesture recognition accuracy, they all have some limitations, because they only use a single type of sensor. Multisensor gesture recognition can perfectly solve these problems by fusing multisensor data, thereby improving the recognition accuracy and recognizing more types of gestures. Plawiak et al. [16] used a DG5 VHand glove device, which consists of five finger flexion sensors and IMU, to identify 22 dynamic gestures, and the recognition accuracy rate reached 98.32%. Lu et al. [18] used the framework of acceleration signal and surface electromyography signal fusion, proposed an algorithm based on Bayesian and dynamic time warping (DTW), and realized a gesture recognition system that can recognize 19 predefined gestures with a recognition accuracy rate of 95.0%. Gesture recognition with multisensor fusion can avoid the limitations of a single sensor, learn from the strengths of multiple approaches, capture the characteristics of each dimension of gestures from multiple angles, and improve the accuracy of gesture recognition.

To date, all these studies are based on gesture recognition on land, and there is no related research on sensor-based gesture recognition underwater. This paper aims to fill this research gap by using a multi-sensor-based gesture recognition approach and developing a new smart data glove that incorporates environmental sensors, five-channel capacitive flexible stretch sensors, and a six-axis IMU (three-axis acceleration meter and three-axis gyroscope) mounted on the back of the hand.

### 2.2. Sensor-Based Gesture Recognition Algorithm

Sensor-based gesture recognition algorithms are generally divided into the following two types: traditional machine learning and deep learning.

Gesture recognition algorithms based on machine learning (ML) include DTW, support vector machine (SVM), random forest (RF), K-means, and K-nearest neighbors [16,19,20,21]. These methods are widely applicable and adaptable to various types of complex gesture data. At present, many researchers have conducted research on the improvement of related algorithms in sensor-based gesture recognition. Although the ML-based gesture recognition method is relatively simple to implement, the number of parameters generated is also lower than that of neural networks, and the requirements for the computing equipment are relatively low. However, with the increase in gesture types and gesture data sequences, the training data required for learning is also increasing. The accuracy and response time of the recognition algorithm will also be affected to a certain extent.

The basic model of deep learning (DL)-based gesture recognition mainly includes the convolutional neural network (CNN) [22], deep neural network (DNN) [23], and recurrent neural network (RNN) methods [24]. The DL model has become the mainstream classification method in gesture recognition due to its excellent performance, high efficiency in extracting data features, and ability to process sequential data. Fang et al. [25] designed a CNN-based SLRNet network to recognize sign language. This method used an inertial-sensors-based data glove with 36 IMUs to collect a user’s arm and hand motion data, and the accuracy can reach 99.2%. Faisal et al. [26] developed a low-cost data glove deployed with flexible sensors and an IMU, and introduced a spatial projection method that improves upon classic CNN models for gesture recognition. However, the accuracy of this method for static gesture recognition is only 82.19%. Yu et al. [27] used a bidirectional gated recurrent unit (Bi-GRU) network to recognize dynamic gestures, realize real-time recognition on the end side (data glove), and reach a recognition accuracy of 98.4%. The limitation of this approach is that it is not possible to only use the smart glove, but external IMUs must be employed on the user’s arm, which can cause discomfort to the user.

The selected model needs to be determined according to the type of task, requirements, and other factors. Due to the complex amphibious environment, the underwater and land environments are different, and the interference to the sensor is entirely different. It is difficult to transmit Bluetooth signals underwater, and it is difficult to send data to the host wirelessly. Therefore, choosing a gesture recognition model suitable for the amphibious environment is essential. This study addresses this gap by proposing a novel amphibious hierarchical gesture recognition (AHGR) model that adaptively switches classification algorithms according to environmental changes (underwater and land) to ensure recognition accuracy in amphibious scenarios. In addition, it is also challenging to ensure accuracy for cross-user and cross-device recognition using a pretrained DL model. Although some studies on gesture recognition across users and in different environments has made some progress [12], they were mainly focused on EMG-based gesture recognition, and there is a lack of research on cross-user gesture recognition using data gloves based on stretch sensors and IMUs. This study, then, introduces the transfer learning framework to the recognition model and proposes a DSN-based gesture recognition transfer model to solve this issue.

## 3. Smart Data Glove and Gesture Set

The following subsections describe in detail the proposed smart data gloves and the predefined gesture set.

### 3.1. Smart Data Glove

**Figure 1:**
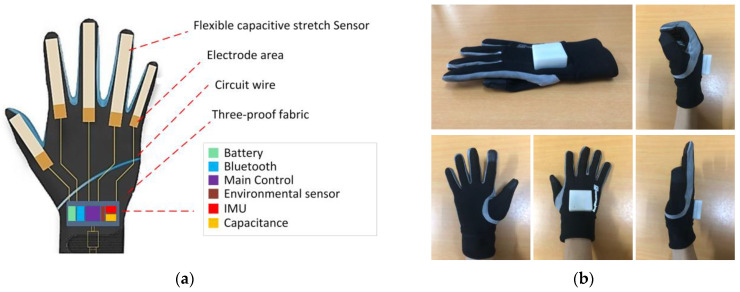
Proposed smart data glove: (**a**) structure diagram, (**b**) appearance.

The smart glove developed in this study is shown in Figure 1. As shown in Figure 1a, the glove uses a five-channel flexible capacitive stretch sensor to collect the bending state of five fingers. The main control module located on the back of the hand is equipped with a Bluetooth communication module for wireless transmission of the collected gesture data, a six-axis IMU (three-axis accelerometer and three-axis gyroscope) for collecting hand spatial motion information, an environmental sensor for inferring the land and underwater environment, a microcontroller to process the collected gesture data and perform some simple computational tasks, and a battery to support electricity energy. The microcontroller used in the smart data glove is the Esp32-S3-DevKitC-1 development board [28]. This microcontroller is equipped with an ESP32-S3-WROOM-1 module, a general-purpose Wi-Fi+ low-power Bluetooth MCU, which has rich peripheral interfaces, powerful neural network computing and signal processing capabilities, and is specially designed for artificial intelligence (AI) and Internet of Things (IoT) market creation. It is equipped with 384 KB of ROM, 512 KB of SRAM, 16 KB of RTC SRAM, and a maximum of 8 MB of PSRAM to meet the experimental requirements. The detailed technical information of the proposed smart data glove is shown in Table 1.

### 3.2. Gesture Set

In the sensor-based gesture recognition technology, according to the characteristics of the stretch sensor and IMU loaded on the data glove, gestures can be divided into dynamic and static gestures according to the characteristics of the activity.

**Figure 2:**
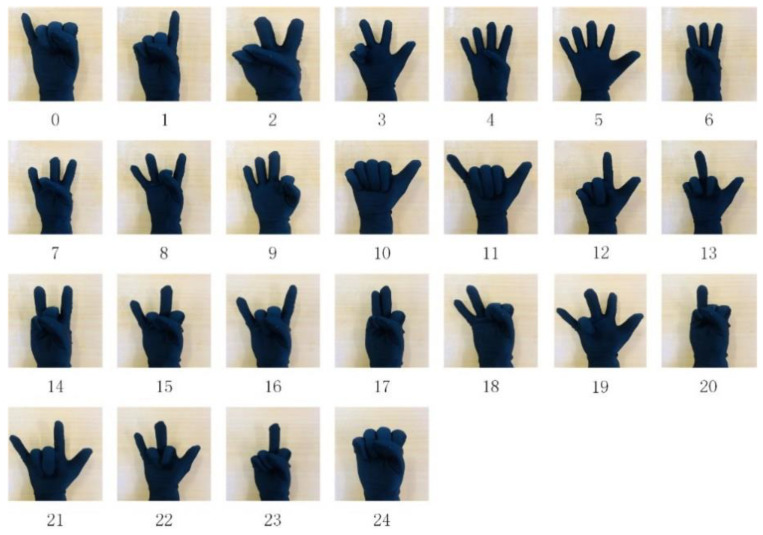
Static gesture set.

Static gestures are defined by the finger bending status. Since there are some difficult-to-operate gestures, some gestures were discarded, and 25 gestures were finally defined, as shown in Figure 2.

**Figure 3:**
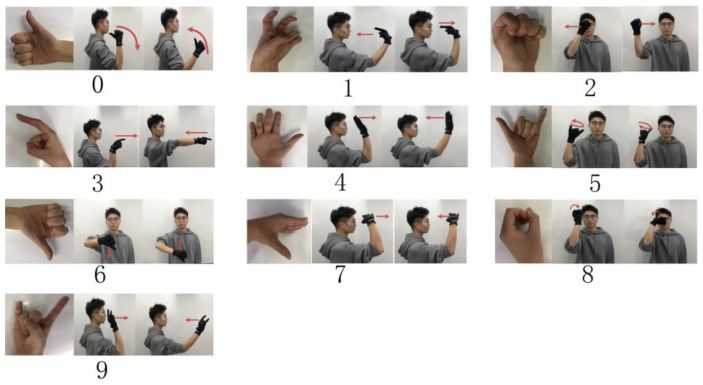
Dynamic gesture set.

Dynamic gestures combine finger bending information (static gesture) with hand motion trajectories to characterize gesture types. We use the signal fluctuation of the motion sensor to distinguish the dynamic and static gestures. At the same time, the definition of the dynamic gesture set refers to the existing gesture sets, such as the sign language gesture set used by deaf–mute patients, and based on the distinguishability, operability, and understandability of the gesture design, 10 dynamic gestures are predefined, as shown in Figure 3.

In the face of different task environments, the gestures’ meanings may differ. Therefore, this research does not assign specific meanings to static and dynamic gestures. It only describes them with serial numbers, where static gestures are assigned with serials from 0–24 and dynamic gestures with 0–9. Thus, users can assign meaning to gestures when dealing with different tasks. In the underwater environment, due to the influence of the water resistance and air pressure, the IMU data will be affected to a certain extent, resulting in data distortion. In contrast, stretch sensor data are very slightly affected by the environment. Secondly, users are easily affected by environmental factors such as the water flow, resulting in movement deviation and incomplete and non-standard gestures. This makes the data collected via IMU vary greatly for the same gesture, making training and testing difficult. Static gestures are less affected by the environment, and they can still be made accurately in an underwater environment. Finally, Bluetooth data are difficult to transmit underwater to the host, and underwater gestures must be recognized on the glove side. Static gesture recognition adopts a lightweight model that can be deployed on a microprocessor with limited computing power, so that static gestures can be recognized directly on the glove side. Based on the consideration of these factors, this study uses static gestures for underwater gesture recognition. The ground environment supports static and dynamic gesture recognition.

## 4. Amphibious Hierarchical Gesture Recognition Model

Due to the differences between underwater and land environments, this study proposes the AHGR model for gesture recognition in amphibious environments with a hierarchical structure. This section describes the details of the proposed AHGR model, including the hierarchical gesture recognition flow, the lightweight stochastic SVD-optimized spectral clustering algorithm for underwater gesture recognition, and the complex SqueezeNet-BiLSTM algorithm for land gesture recognition.

### 4.1. Hierarchical Gesture Recognition Flow

Affected by the underwater environment, it is difficult for users to make precise dynamic gestures underwater. The IMU signal will be greatly disturbed underwater, affected by water pressure, resistance, water flow, etc. Static gestures have no complex spatial motion, relying only on stretch sensor data to represent the gesture state information. Additionally, stretch sensors are less affected by the underwater environment. Thus, underwater gesture recognition only considers static gesture recognition using stretch sensor data. And since gesture recognition needs to be performed directly on the glove end in an underwater environment, choosing a recognition algorithm model with less recognition latency and less computing power requirements is necessary to ensure adequate gesture recognition performance in an underwater environment. Therefore, this study proposes a lightweight stochastic SVD-optimized spectral clustering algorithm to recognize underwater static gestures.

In the land environment, both static and dynamic gesture recognition are relatively easy to implement and acquire. There are still some challenges regarding dynamic gesture recognition on land. Although there is no interference from the water environment, the user will inevitably tremble to a certain extent when making gestures, which will cause fluctuations in sensor (IMUs) data and affect the recognition accuracy. The dynamic gesture recognition problem is a placement-independent problem with strong temporal characteristics, and a model capable of deep feature extraction in temporal and spatial dimensions is required. Thus, this study adopts the method of multisensor data fusion and proposes a complex SqueezeNet-BiLSTM algorithm for dynamic gesture recognition on land to ensure the effectiveness, robustness, and accuracy of the recognition results.

**Figure 4:**
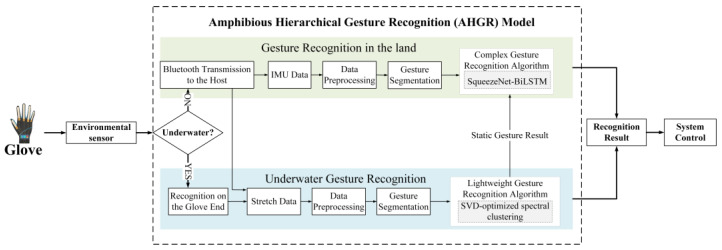
Amphibious hierarchical gesture recognition (AHGR) model.

As shown in Figure 4, the detailed amphibious gesture recognition process of the AHGR model is as follows: The AHGR model first determines the recognition environment based on environmental sensors. The environmental sensor used in the AHGR model is a barometer sensor. According to the principles of hydrostatic pressure, when the air pressure sensor value is greater than the local standard atmospheric pressure plus 0.98 kpa (water depth is greater than 0.1 m), the current environment is underwater; otherwise, it is judged to be a land environment. If it is underwater, the AHGR model will switch to underwater gesture recognition and use the proposed lightweight stochastic SVD-optimized spectral clustering algorithm to recognize static gestures on the glove side. If it is on land, the AHGR model will first switch to land gesture recognition and determine the dynamic and static gestures through the fluctuations in the IMU data. If it is a static gesture, the land gesture recognition will directly output the result of the static gesture recognized using the lightweight stochastic SVD-optimized spectral clustering algorithm. If it is a dynamic gesture, land gesture recognition will use the SqueezeNet-BiLSTM algorithm to recognize dynamic gestures using multisensor data and encoded static gesture recognition results. The recognition results can be used to interact with or control devices in the IoT environment.

### 4.2. Stochastic SVD-Optimized Spectral Clustering Algorithm

The spectral clustering algorithm is an algorithm evolved from graph theory [29]. Its main idea is to regard all data as points in the space, connect them with edges in the graph, calculate the weight by calculating the distance from the point to the edge, and finally realize clustering according to the weight. Although the spectral clustering algorithm can complete the clustering of high-dimensional data, the spectral clustering algorithm relies too heavily on the Laplacian matrix to complete the eigen decomposition. The calculation process requires extremely high space complexity and time complexity, and with the increase in data volume, the complexity also increases exponentially, seriously affecting the practical applications. Therefore, this study introduces the stochastic SVD [30] algorithm to accelerate the spectral clustering algorithm and reduce the computational cost.

SVD is a matrix decomposition method widely used in pattern recognition to reduce dimensions and solve ranks. The main process is to establish the connection between the large matrix and the small matrix and estimate the SVD result of the large matrix through the SVD decomposition result of the small matrix. This study considers using a stochastic SVD [31] algorithm. In this algorithm, an orthogonal matrix is established first and used as an orthogonal basis for the low-rank estimation of the original matrix. At the same time, the original matrix is projected to the subspace, the matrix formed in the subspace is subjected to SVD, and the decomposition result is mapped back to the original space. The detailed process is as follows:

Let the original matrix be $W\in R^{n\times n}$. First, select a standard Gaussian random matrix Ω of n×(k+p), where *k* is the dimension of the low-rank estimate, and *p* is the oversampling parameter, so that the rank of the random subspace is slightly larger than *k*. Let *Z* = *W*Ω, and then find an orthogonal matrix $Q\epsilon R^{n\times k}$ through QR decomposition to let $Z=QQ^{T}Z$. Map the original matrix *W* to the subspace with *Q* as the orthogonal basis, and obtain

(1) $$B=Q^{T}WQ,$$

For the SVD decomposition of B, obtain

(2) $$B=VMV^{T},$$

Then, the k-rank estimation of the original matrix *W* is obtained as

(3) $$W\approx Q{BQ}^{T}=QVMV^{T}Q^{T},$$

Therefore, the estimated eigenvector of *W* is *U* = *QV*. The stochastic SVD algorithm avoids direct SVD decomposition of large matrices by mapping high-dimensional matrices to low-dimensional subspaces. Hence, the information on the original matrix is almost completely preserved. The stochastic SVD-optimized spectral clustering algorithm is shown below as Algorithm 1.
**Algorithm 1:** SVD-optimized spectral clustering**Input**: $X=\{x_{1},x_{2},\ldots ,x_{n}\},x_{i}\in R^{N}$ **Output**: Clustering result of $x_{1},x_{2},\ldots ,x_{n}$ **for** i, j = 1, …, n:
$\;\;\;\;s\left(x_{i},x_{j}\right)\leftarrow exp\left(\frac{*-d{\left(x_{i},x_{j}\right)}^{2}}{2\sigma^{2}}\right)$
${\;\;\;\;A}_{ij}=s\left(x_{i},x_{j}\right)$ **end** [u,s,v] = Randomized_SVD(A) # u, v is the left and right singular vector matrix of A # s is the singular value matrix of A, $s=diag\left(\sigma_{1},\sigma_{2},\ldots ,\sigma_{n},\right)$ $U\leftarrow \left\{u_{1},u_{2},\ldots ,u_{l}\right\}\in R^{n\times l}$, where ui is the i-th vector of u $y_{i}\in R^{l},i=1,2,\ldots ,n$ is the i-th row vector of matrix U $C_{1},C_{2},\ldots ,C_{k}\leftarrow Kmeans(y_{i})$ Create mapping $x_{i}\in R^{N}⊢y_{i}\in R^{l},i=1,2,\ldots ,n$ Output the clustering results of $x_{1},x_{2},\ldots ,x_{n}$

### 4.3. SqueezeNet-BiLSTM Algorithm

**Figure 5:**
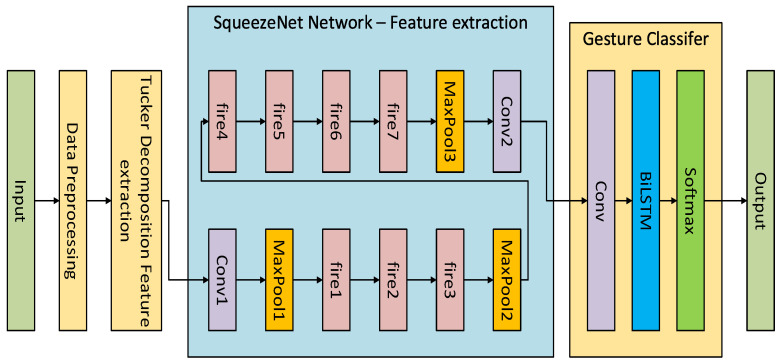
Structure diagram of SqueezeNet-BiLSTM algorithm.

The proposed SqueezeNet-BiLSTM gesture classification algorithm first uses the Tucker decomposition algorithm to reduce the dimensionality and extract features of the preprocessed gesture data. After that, the SqueezeNet [32] network is used to extract in-depth data features and combined with the Bi-LSTM [33] network to extract the time series features of the gesture data to ensure the robustness of the gesture recognition model and improve the recognition accuracy. Tucker [20] decomposition is a high-dimensional data analysis method, especially suitable for dimensionality reduction and feature extraction of multidimensional data. It decomposes higher-order tensors into products of core tensors and some modality matrices. In this process, the dimensionality reduction of the data can be achieved by retaining the principal components of the core tensor, thereby removing irrelevant information and noise. The SqueezeNet [24] network adopts the idea of compression and expansion. Compared with the traditional convolutional neural network, it reduces the model parameters while ensuring the gesture recognition accuracy. A Bi-LSTM network, through the stacking of two layers of LSTM structure, solves the limitation that LSTM can only predict the output of the next moment based on the timing information of the previous moment. It can better combine the context for output and more effectively utilize the input gesture data’s forward and backward feature information. The structure diagram of the proposed SqueezeNet-BiLSTM algorithm is shown in Figure 5.

The gesture recognition process of the SqueezeNet-BiLSTM model is as follows: For the gesture data collected by the smart data glove, the scale of the original sensor data is adjusted to a uniform length through operations such as sliding window, filter processing, standardization, normalization, data length normalization, and Turker decomposition [34]. The processed gesture feature data are input into the SqueezeNet network to obtain the corresponding feature vector through the multilayer convolution module, fire module, and maximum pooling layer, and then, the time series features are extracted from the gesture data through the BiLSTM network, and finally through the SoftMax to complete the gesture classification.

## 5. DSN-Based Gesture Recognition Transfer Model

During gesture recognition, the collected gesture data from the data gloves are subject to variations due to different users and different data gloves, leading to discrepancies that result in reduced recognition accuracy when incorporating new users or new data gloves into the recognition system. Employing user-specific model training during recognition requires substantial data from diverse users. While this approach may yield personalized gesture recognition models tailored to the unique characteristics of each user, it can potentially compromise the user experience for new users. Leveraging transfer learning facilitates the adaptation of existing gesture recognition models to acquire the distinctive gesture data features associated with new users and new data gloves. This approach enables the preservation of the intrinsic gesture recognition domain features while concurrently acquiring domain-specific features from the new context, thereby enhancing the recognition efficiency of the source model when confronted with novel data. Therefore, this study presents a novel DSN-based [35] gesture recognition transfer model, leveraging the principles of transfer learning. By collecting a small but representative dataset from the new domain, this model facilitates the transfer of the gesture recognition model, ensuring its effectiveness in accurately recognizing new data and enhancing the overall user experience.

### 5.1. Domain Separation Networks

Considering the inherent differences in gesture data among various users and different data gloves, it is acknowledged that the data space for gesture data is not entirely congruent. However, it is observed that certain common features exist alongside the distinct characteristics that are specific to each data domain. A transfer learning methodology utilizing DSN is considered to address this. This approach aims to uncover shared feature representations across users and data gloves while capturing domain-specific features simultaneously. During the transfer process, the source domain’s private features are discarded, while the shared features are preserved, thereby ensuring the successful migration of the model.

**Figure 6:**
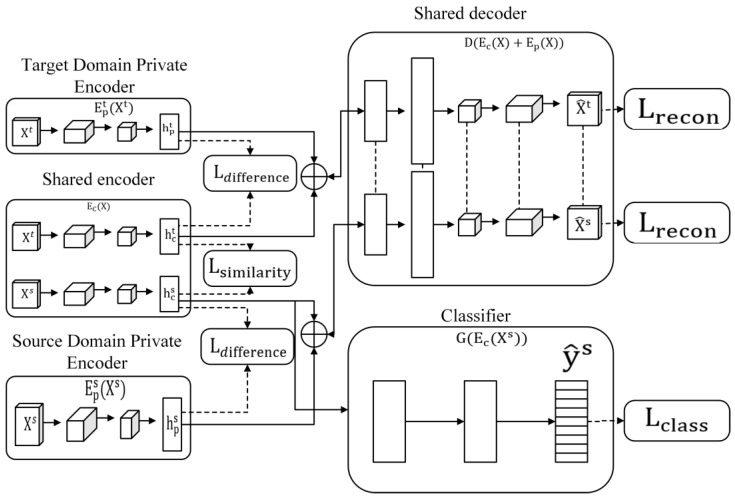
DSN structure diagram.

The main work of DSNs [35] is divided into two parts: extracting common features of different domains and using common features for migration. The obtained DSN structure is shown in Figure 6.

A DSN is a “Decoder-Encoder” structure, which can be divided into five parts:

1. Target Domain Private Encoder $E_{P}^{t}(X^{t})$: Used to extract private features of the target domain.
2. Source Domain Private Encoder $E_{P}^{s}(X^{s})$: Used to extract private features of the source domain.
3. Shared Encoder $E_{c}(X)$: Used to extract the common features of the source and target domains.
4. Shared Decoder $D(E_{c}(X)+E_{p}(X))$: Used to decode samples composed of private features and shared features.
5. Classifier $G(E_{c}\left(X^{s}\right))$: The source domain samples are classified during training, and the classification is completed directly on the target domain when the training is completed.

The overall work of the DSN is based on the original gesture recognition model structure, the model is used as an encoder, and the overall training goal is to minimize the difference loss $L_{difference}$:

(4) $$L_{difference}={\left\|{H_{c}^{s}}^{T}H_{p}^{s}\right\|}_{F}^{2}+{\left\|{H_{c}^{t}}^{T}H_{p}^{t}\right\|}_{F}^{2}$$

$L_{difference}$ calculates the similarity between $h_{c}^{s}$ and $h_{p}^{s}$ and $h_{c}^{t}$ and $h_{p}^{t}$. When $h_{c}^{s}$ = $h_{p}^{s}$ and $h_{c}^{t}$ = $h_{p}^{t}$, $L_{difference}$ is the largest, and when $h_{c}^{s}$ and $h_{p}^{s}$ are orthogonal (that is, completely different) and $h_{c}^{t}$ and $h_{p}^{t}$ are orthogonal, $L_{difference}$ is the smallest. Therefore, the purpose of completely separating $h_{c}^{s}$ from $h_{p}^{s}$ and $h_{c}^{t}$ from $h_{p}^{t}$ can be achieved by minimizing $L_{difference}$.

While ensuring that $h_{c}^{s}$ and $h_{p}^{s}$ and $h_{c}^{t}$ and $h_{p}^{t}$ are completely separated, it is necessary to ensure that $h_{c}^{s}$ s and $h_{c}^{t}$ can be transferred, meaningthat it is necessary to improve the similarity between the two, that is, to reduce the similarity loss $L_{similarity}$:

(5) $$L_{similarity}=\frac{1}{{\left(N^{s}\right)}^{2}}\sum_{i,j=0}^{N^{s}}k(h_{ci}^{s},h_{cj}^{s})-\frac{1}{N^{s}N^{t}}\sum_{i,j=0}^{N^{s},N^{t}}k(h_{ci}^{s},h_{cj}^{t})+\frac{1}{{\left(N^{t}\right)}^{2}}\sum_{i,j=0}^{N^{t}}k(h_{ci}^{t},h_{cj}^{t})$$

When the similarity loss $L_{similarity}$ is the smallest, $h_{c}^{s}$ and $h_{c}^{t}$ can be made the most similar or even become the same distribution. When the two distributions are similar, the classifier that is effective on $h_{c}^{s}$ can also work on $h_{c}^{t}$. While meeting the above conditions, it is also necessary to complete the measurement of the source domain data and perform target domain data assurance. Using the “encoder-decoder” structure, set the reconstruction loss $L_{recon}$:

(6) $$L_{si\_mse}(x,\hat{x})=\frac{1}{k}{\left\|x-\hat{x}\right\|}_{2}^{2}-\frac{1}{k^{2}}{\left([x-\hat{x}]\cdot 1_{k}\right)}^{2}$$

(7) $$L_{recon}=\sum_{i=1}^{N_{s}}L_{si\_mse}(x_{i}^{s},{\hat{x}}_{i}^{s})+\sum_{i=1}^{N_{t}}L_{si\_mse}(x_{i}^{t},{\hat{x}}_{i}^{t})$$

After extracting the shared features and their respective private features of the source domain and target domain samples, it is still necessary to classify the samples and set the classifier loss function $L_{task}$. After minimizing $L_{similarity}$, the distribution of the shared part of the source domain and the target domain is approximated. The classifier is effective in the common part of the source domain while ensuring that the common part of the target domain is also effective. Therefore, it only needs to use the labeled source domain data to train the classifier.

(8) $$L_{task}=-\sum_{i=0}^{N_{s}}y_{i}^{s}\cdot log{\hat{y}}_{i}^{s}$$

### 5.2. The Structure of the Gesture Recognition Model

**Figure 7:**
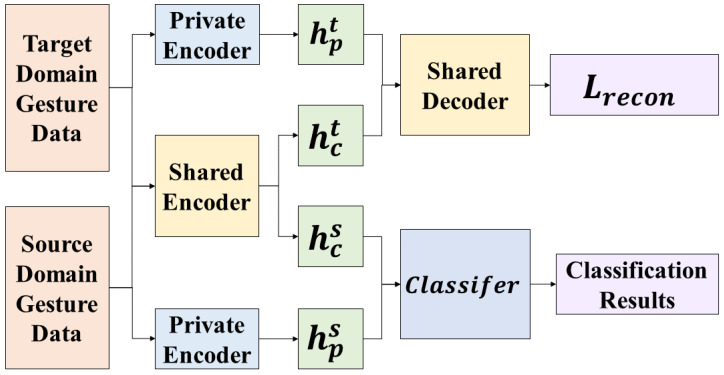
DSN-based small-sample gesture recognition transfer model.

According to the DSN structure and basic principles, and based on the gesture recognition process, the small-sample gesture recognition transfer model proposed in this study is shown in Figure 7.

**Figure 8:**
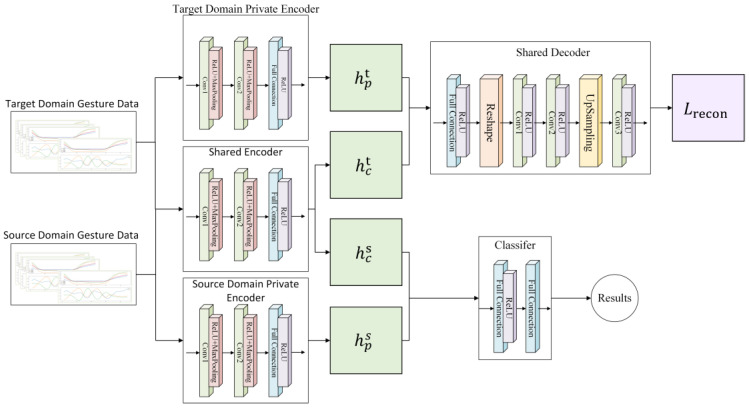
Proposed DSN network structure diagram.

The network recognition process is as follows: The labeled source domain gesture data are processed using private encoders and shared encoders to extract private features and shared features, respectively. Similarly, the unlabeled target domain gesture data are processed using private encoders and shared encoders to extract private features and shared features separately. By performing the computations outlined in Equations (4) and (5), the difference loss $L_{difference}$ and similarity loss $L_{similarity}$ are obtained. The shared features from the source and target domains, along with the target domain’s private features, are fed into the shared decoder. This process involves the computations specified in Equations (5) and (6), resulting in the reconstruction loss $L_{recon}$. Furthermore, a classifier $L_{task}$ is constructed using the shared features from the source domain and the corresponding data labels. This entire procedure is repeated iteratively to minimize the overall loss function $L_{task}+\alpha L_{recon}+\beta L_{difference}+\gamma L_{similarity}$, where *α*, *β*, and *γ* are hyperparameters controlling the respective loss terms. Ultimately, the obtained classifier is utilized for recognizing gesture data collected from the target domain, i.e., new users with new data gloves. The network structure of the encoder and decoder is shown in Figure 8.

For the encoder part, we use a two-layer convolution structure to encode the gesture data. The first-layer convolution kernel size is set to three and passed through the ReLU layer to accelerate model convergence. At the same time, a maximum pooling layer with a kernel size of two is used to alleviate the convolution layer’s sensitivity to positional relationships. The second-layer convolution kernel size is five in order to capture the data correlation characteristics of different areas. It then adopts a similar ReLU layer and maximum pooling layer, and then accesses the coding features obtained by the fully connected layer output operation.

For the shared decoder part, we first use the fully connected layer to decode the private features and public features and use the Reshape unit to modify the output of the fully connected layer to the size corresponding to the convolutional neural network. Then, we use two layers of convolution and ReLU layers with a convolution kernel of five and a UpSampling unit for deconvolution to restore the data. Finally, the restored data are operated through the convolution and ReLU layers to obtain the reconstruction loss $L_{recon}$.

## 6. Experimental Results and Analysis

This section will discuss the gesture data collection, experiments, and results to verify the effectiveness of the AHGR model proposed in this study.

### 6.1. Data Collection

Based on the amphibious environment, this study will collect and build hand gesture datasets in land and underwater environments. The gesture data collection setup is shown in Table 2.

The land environment’s gesture dataset includes dynamic and static gesture data. A total of 20 volunteers participated in the data collection experiments. During the data collection, the volunteers were asked to wear a data glove on their right hand and maintain a stable standing posture. After starting the gesture collection, volunteers had to make corresponding predefined dynamic and static gestures, and each gesture lasted for ten minutes. The land gesture dataset collected a total of 250,000 sets of static gesture data and 100,000 sets of dynamic gesture data, and each set of data comprises 60 data points, which is the window size.

**Figure 9:**
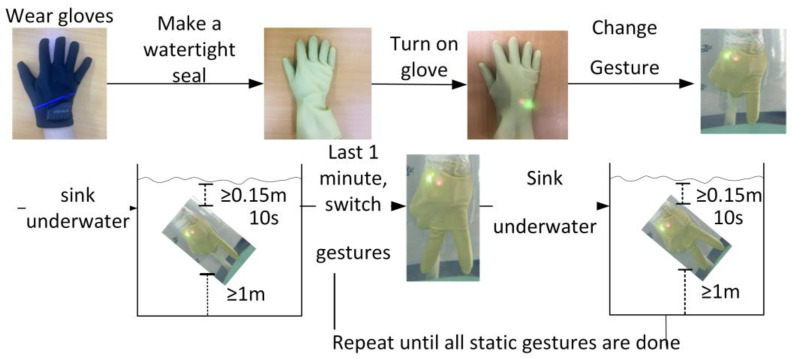
Underwater gesture data collection flow diagram.

The underwater gesture dataset is defined and constructed for the static gesture set, and the data collection flow diagram is shown in Figure 9. The underwater gesture data collection process is as follows: First, simulate the underwater environment and use a water-filled pool. Second, 20 volunteers put the smart data glove on their right hand, then put on a thin nitrile glove to make it waterproof. Third, volunteers put their hands into the water-filled pool, make the corresponding gesture, and then turn on the data glove’s power. The fingers of the hand should be at least 0.15 m away from the bottom of the pool, and the elbow should be at least 0.5 m away from the water’s surface. For each gesture, the volunteers had to remain underwater for at least 1 min. After a gesture data collection process is completed, the glove must be connected to the computer to export the gesture data saved on the glove side. According to the static gesture set, repeat the above steps until all 25 predefined static gesture data are collected. The underwater gesture dataset collected a total of 25,000 sets of static gestures, and each set of data comprises 60 data points.

### 6.2. Evaluation of the Stochastic SVD-Optimized Spectral Clustering Algorithm

**Figure 10:**
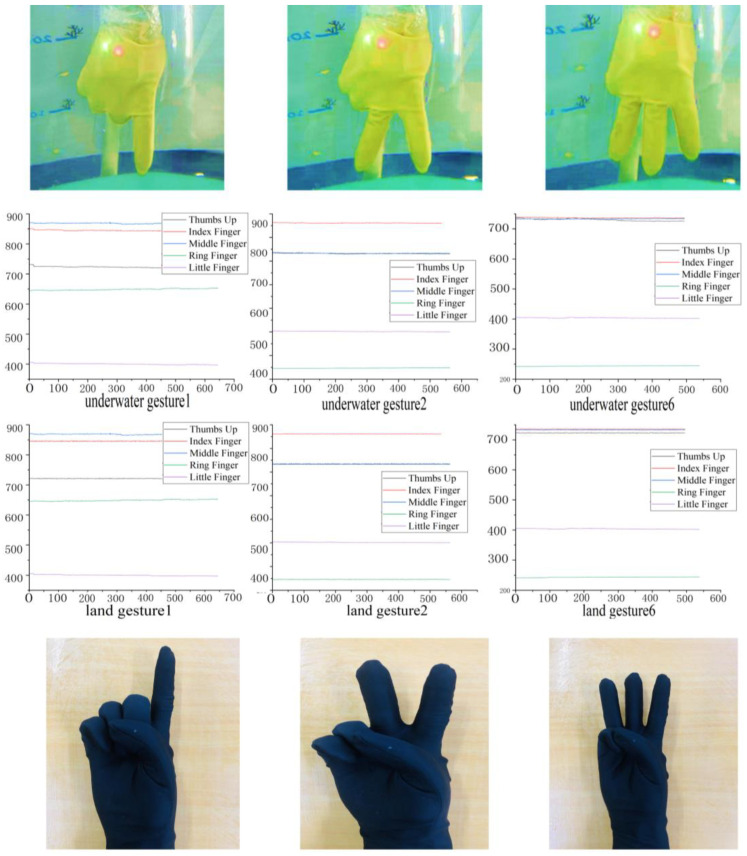
Comparison of static gesture data collected from underwater and land.

**Figure 11:**
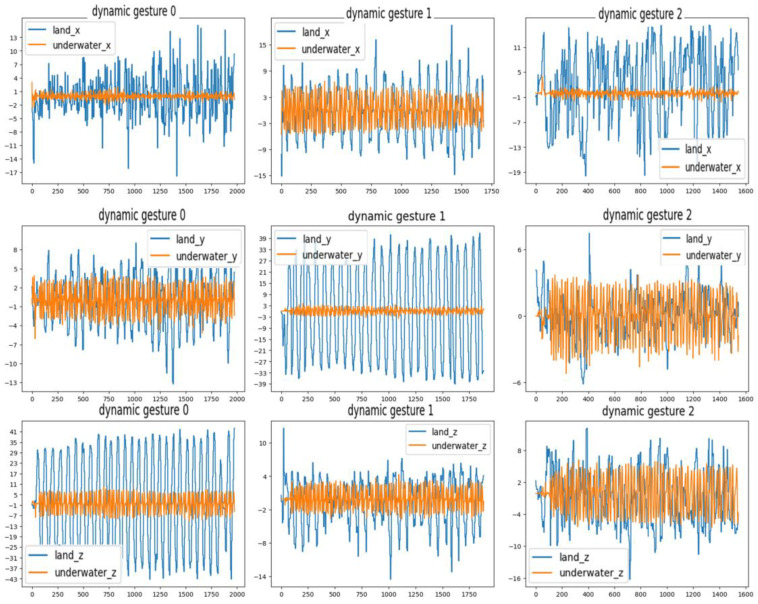
Comparison of dynamic gesture data collected from underwater and land.

Due to the usage of a static gesture set for underwater gestures, this research focuses solely on the gesture characteristics conveyed by the stretch sensors in the underwater data, while disregarding the data from the IMU. The comparison between the collected underwater gesture data and the corresponding land-based gesture data is illustrated in Figure 10 and Figure 11. As shown in Figure 10, the upper part of the figure represents the underwater gesture data, while the lower part represents the gesture data captured on land. The figure displays three gestures, numbered 1, 2, and 6, from the predefined static gesture set depicted in Figure 2. As shown in Figure 11, the blue curve represents the gesture data collected underwater, and the orange curve represents the gesture data collected on land. A total of three dynamic gesture data points are compared in Figure 11, namely, dynamic gestures 0, 1, and 2 from the predefined dynamic gesture set depicted in Figure 3.

As illustrated in Figure 10, after undergoing gesture preprocessing and standardization normalization, the underwater gesture data captured by the stretch sensors exhibit similarity to the land-based gesture data collected by stretch sensors. The signal variations caused by the water pressure on the stretch sensors are found to be less than 1%. As illustrated in Figure 11, the dynamic gesture data show huge differences between underwater and on land, which can make pretrained dynamic gesture models difficult to use underwater. The above comparative results verify the feasibility of using static gestures underwater and the difficulty of using dynamic gestures. Moreover, since the underwater environment has little impact on the gesture data, the verification of underwater gesture recognition algorithms (stochastic SVD-optimized spectral clustering algorithm) can use on-land static gesture data as a reference.

A total of 25 static gesture data samples from 10 individuals were collected for experimentation. The collected data underwent preprocessing, normalization, and standardization procedures, with the application of a sliding window filtering technique to eliminate noise. Feature vectors were extracted from each gesture sample, and the extracted data were inputted into the stochastic SVD-optimized spectral clustering algorithm. The recognition accuracy and recognition time were recorded and compared with the performance of classic classifiers such as SVM, K-NN, and multilayer perceptron (MLP). The comparative results are summarized in Table 3.

The experimental validation revealed that the gesture recognition algorithm employed in this study achieved an average recognition accuracy that was approximately 7% higher than for SVM, 3% higher than for K-NN, and around 2% higher than for spectral clustering. Furthermore, the inference time and training time of the proposed algorithm were comparatively shorter than those of the other algorithms. These results provide empirical evidence of the effectiveness of the adopted stochastic SVD-optimized spectral clustering algorithm for underwater gesture recognition.

### 6.3. Evaluation of the SqueezeNet-BiLSTM Algorithm

To evaluate the proposed SqueezeNet-BiLSTM algorithm, this study conducted comparative experiments on several sensor-based deep learning gesture recognition algorithms, including convolution neural network (CNN)-LSTM, BiLSTM, CNN-LSTM, and SqueezeNet-LSTM.

The CNN-LSTM [36] network is a classic DL network model. It uses a CNN to extract the features of gestures in the spatial dimension and LSTM to extract the features of gesture data in the time dimension. The BiLSTM [37] network uses bidirectional LSTM network units to realize the two-way feature in the time dimension of gesture data extraction. The CNN-BiLSTM network [38,39] combines the classic CNN and BiLSTM to compare and verify the impact of the SqueezeNet network architecture on the accuracy of gesture recognition. The SqueezeNet-LSTM network connects the SqueezeNet network and the LSTM network to show the characteristics of the bidirectional time feature extraction of the gesture data via the bidirectional LSTM network.

**Figure 12:**
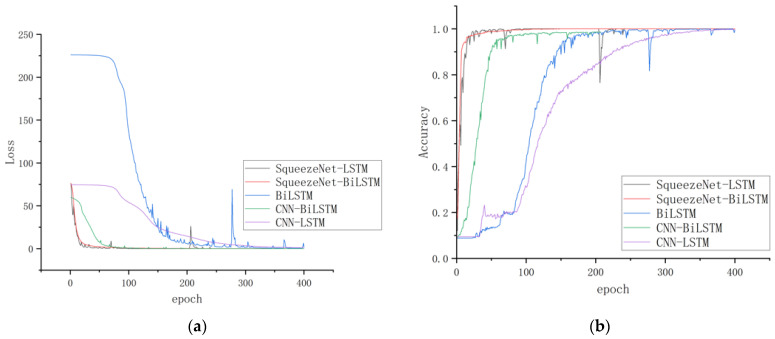
Loss and accuracy curve for selected algorithm: (**a**) loss curve; (**b**) accuracy curve.

The dataset collected in Section 6.1 is used to train the model. The ratio of training and testing sets is 7:3. The window size is set up to be 60. The loss and accuracy curves of the five selected algorithms are shown in Figure 12. As the epoch increases, the loss rate of the training model gradually approaches 0, and the accuracy rate approaches 1. Although the trends of all training models tend to be consistent in the end, the loss and accuracy curve of the SqueezeNet-BiLSTM algorithm is smoother, and it converges faster than those of the other four selected algorithms, which shows that the performance of the SqueezeNet-BiLSTM algorithm is more suitable for the current situation. The performance results of the proposed SqueezeNet-BiLSTM algorithm and the other four selected algorithms are shown in Table 4.

According to the above experimental results, the recognition accuracy of the gesture recognition based on the BiLSTM network is the worst compared with other algorithms and can only reach 92.3%. Its network structure only pays attention to the information characteristics of the gesture sequence in the time dimension, ignoring the character of the gesture data in the spatial dimenstion, and the recognition accuracy is relatively low. The recognition accuracy of the gesture recognition algorithm based on the CNN-LSTM network structure and the CNN-BiLSTM network structure is higher than that of the gesture recognition algorithm based on the BiLSTM network. This is because its network structure fully integrates the characteristics of CNN and LSTM networks and fully extracts the attributes of gesture data in various dimensions. The recognition accuracies obtained by the CNN-LSTM and the CNN-BiLSTM network are close. The reason is that the two network structures are similar, and the difference mainly lies in the Bi-LSTM network structure used by the latter.

Compared with the other four selected classification algorithms, the gesture recognition algorithm based on the SqueezeNet-BiLSTM network proposed in this study has the best recognition accuracy, and its recognition accuracy, precision, recall, and F1 score reach 98.94%, 97.34%, 98.21%, and 97.21%, respectively. Its training time and inference time are at a medium level compared with the state-of-the-arts algorithms. This is an acceptable result, because although SqueezeNet is a lightweight convolutional neural network, whose training time and inference time are usually short, when the BiLSTM layer is connected behind SqueezeNet, as the complexity of the model increases, the recognition accuracy increases, and the training time and inference time inevitably increase.

### 6.4. Evaluation of DSN-Based Gesture Recognition Transfer Model

**Figure 13:**
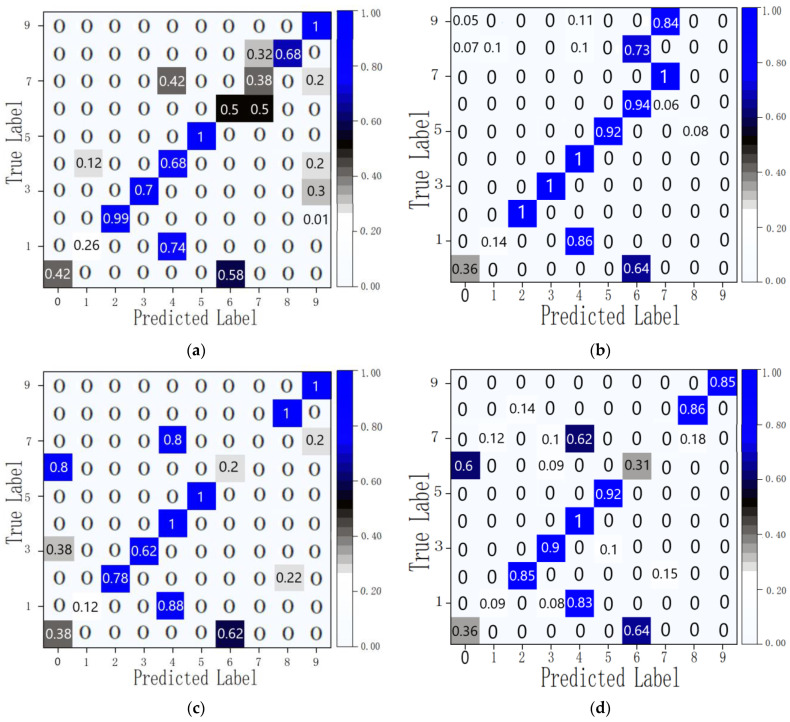
Confusion matrix of new users’ recognition: (**a**) UserA; (**b**) UserB; (**c**) UserC; (**d**) UserD.

The experiment employed the gesture data of two volunteers to validate the efficacy of the proposed DSN-based gesture recognition transfer model. The experiment randomly selected four volunteers as UserA, UserB, UserC, and UserD. Their gesture data were excluded from the collected dataset, and the remaining data were utilized to train the SqueezeNet-BiLSTM source model. Following the completion of training, the model was tested by inputting the gesture data of these four users and the remaining data. The obtained average recognition accuracy is presented in Table 5, while the confusion matrix of users A and B is shown in Figure 13. The outcomes reflected in Table 5 underscore the substantial dissimilarities among the gesture data of different users, with the source gesture recognition model failing to extract the distinctive features of the novel users’ gesture data, leading to a diminished accuracy in recognizing new users’ gestures.

Figure 13 demonstrates that certain gesture recognition accuracies, such as gestures 0, 1, 6, and 7, are notably low. Gesture 0 and gesture 6 are often misrecognized for each other. This may be because the finger bending state is the same in the two gestures, and the hand movements are also similar. This leads to mutual misrecognition when user actions are not very standardized. Gestures 1 and 7 are always recognized as gesture 4. This may be due to the similar hand movements of these gestures and the non-standard bending of the user’s fingers. In Figure 13, some special cases arise; in the test results of User B, gestures 8 and 9 show recognition problems, which may be caused by non-standard bending movements of the user’s fingers or ill-fitting gloves. Since our gloves only come in one size, people with small hands cannot fit the gloves perfectly when wearing them, making it difficult to obtain accurate stretch sensor data, ultimately leading to inaccurate recognition. For other relatively small identification problems, these can be attributed to variances in personal hand size, movement patterns, and sensor data from the glove, resulting in significant disparities between certain gesture data and the data employed during training. To avoid these problems, we will first perform bending and stretching calibration in the early stage of gesture recognition to minimize recognition errors caused by palm size. Secondly, in the data preprocessing stage, filtering algorithms are used to reduce data noise and then put through data normalization, as well as data up-sampling and down-sampling, to reduce dynamic gesture recognition errors caused by personal hand movement habits. Although a series of measures have been taken to ensure the accuracy of identification, everyone’s behavioral habits still vary greatly. In practical environments, it is still difficult to obtain good recognition accuracy using untrained data.

**Figure 14:**
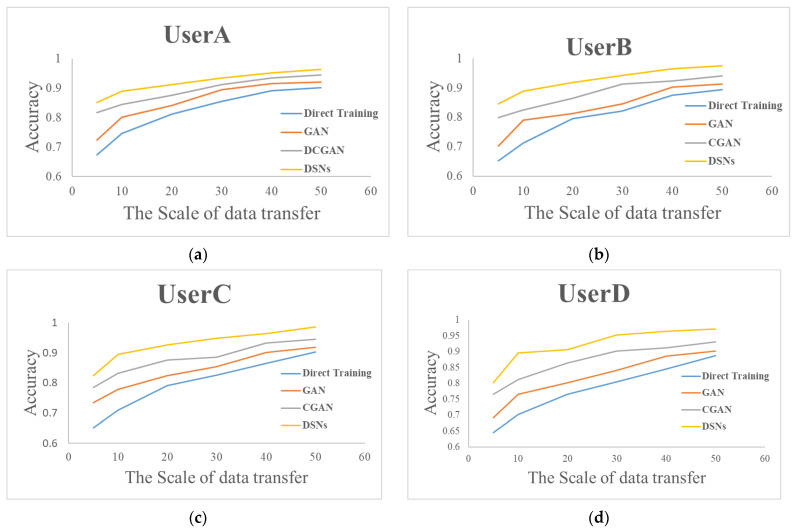
Transfer experiment of new user gesture recognition based on DSNs. (**a**) UserA; (**b**) UserB; (**c**) UserC; (**d**) UserD.

The experiment performed a model transfer test regarding small-sample data, using gesture data of varying scales. Specifically, the experiments collected samples of 5, 10, 20, 30, 40, and 50 instances for each gesture category. To verify the superiority of our proposed -DSNbased gesture recognition transfer model, we also selected several state-of-the-art transfer learning models for comparison, including generative adversarial network (GAN)- [40] and conditional generative adversarial networks (CGAN)-based [41] transfer learning models. The transfer process involved utilizing our proposed DSN-based gesture recognition transfer model and selected state-of-the-art transfer learning models, with incremental updates applied to enhance the model’s performance. Subsequently, the experiment conducted tests using the gesture data of UserA, UserB, UserC, and UserD to evaluate the recognition accuracy of the transferred gesture recognition model. The results depicting the recognition accuracy for each user are illustrated in Figure 14.

As shown in Figure 14, it can be observed that the recognition accuracy for new users increases with the growth of the data scale. During data transfer training with the same sample size, the accuracy of the proposed DSN-based gesture recognition transfer model is significantly better than the state-of-the-art algorithms. When using the novel DSN gesture recognition transfer model in the target domain, the model effectively extracts the domain-generalizable features from the source domain data and applies them to the target domain. As a result, the recognition accuracy is significantly improved compared with direct training when conducting small-scale data transfer training. Therefore, new users only need to provide a small amount of training data to ensure the accuracy of the recognition model, thereby effectively enhancing the user experience.

## 7. Conclusions and Future Work

This study developed a smart data glove with five-channel flexible capacitive stretch sensors, accelerometers, and gyroscopes for gesture recognition in an amphibious environment. To ensure recognition accuracy, this study also proposed a novel AHGR model, which can adaptively change the gesture recognition model to adopt an amphibious environment. This model contains two classification algorithms, the SqueezeNet-BiLSTM algorithm for land gesture recognition and the stochastic SVD-optimized spectral clustering algorithm for underwater gesture recognition. The accuracy of the SqueezeNet-BiLSTM algorithm and the stochastic SVD-optimized spectral clustering algorithm can reach 98.94% and 98.35%, respectively. This study also introduces a DSN-based gesture recognition transfer model, so that new users and new devices only need small-scale data transferring and training to ensure that the recognition accuracy reaches 94%.

In future work, we plan to conduct more professional underwater hand gesture testing, such as hiring divers to test in deeper water. We also plan to develop a waterproof smart data glove that can be used directly underwater and add an acoustic modem to transmit gesture data wirelessly. In addition, we plan to analyze the energy consumption of different models running on the gloves and optimize the design model to reduce energy consumption while ensuring high accuracy.

## Figures and Tables

**Table 1:** Detailed technical information of the proposed smart data glove.

Indicator Name	Parameter
Stretch range	0–50%
Minimum trigger strain	0.05%
Response time	15 ms
Stretch fatigue	>100,000 times
Data transmission	Bluetooth wireless data transmission.
Data collection frequency	50 Hz
Microcontroller	Esp32-S3-DevKitC-1
Battery capacity	500 mah (when we deploy the proposed lightweight model on the glove side and conduct static gesture recognition and output signals in real time via Bluetooth. Through experimental testing, the battery power can last for 5–6 h.)
Battery type	Lithium polymer battery
Environmental sensor	Barometer Senor QMP6988
IMUs	MPU6050 (3-axis accelerometer and gyroscope)

**Table 2:** Gesture data collection setup.

Parameters	On Land	Underwater
Gesture Type	25 static gestures and 10 dynamic gestures	25 static gestures
Data collected	3-axis acceleration data, 3-axis gyroscope data, and 5-channel stretch data	5-channel stretch sensor data
Data collection devices	Developed smart data glove and used Bluetooth to transfer data to Android devices	Esp32-S3-DevKitC-1
Sensors	Accelerometer, gyroscope, and 5-channel flexible capacity stretch sensors	Smart data glove built-in 5-channel flexible capacity stretch sensors
Sampling rate	50 HZ (0.02 s per reading)	50 HZ (0.02 s per reading)
Time duration	10 min for each gesture	10 min for each gesture
Environment	20 volunteers wear smart data gloves on land	20 volunteers wear smart data gloves and put them under water
File format for storing data	.txt file	.txt file

**Table 3:** The performance results of different classification algorithms for underwater gestures.

	SVM	K-NN	Spectral Clustering	MLP	Stochastic SVD-Optimized Spectral Clustering
Person 1	0.9857	0.9667	0.9519	0.9651	0.9759
Person 2	0.9333	0.9784	0.9484	0.9545	0.9828
Person 3	0.9333	0.9667	0.9324	0.9456	0.9865
Person 4	0.9996	0.9568	0.9349	0.9464	0.9794
Person 5	0.9655	0.9935	0.9724	0.9652	0.9827
Person 6	0.9666	0.9667	0.9282	0.9413	0.9863
Person 7	0.8275	0.9382	0.9873	0.9613	0.9827
Person 8	0.9667	0.9348	0.9932	0.9734	0.9932
Person 9	0.7586	0.9655	0.9923	0.9426	0.9793
Person 10	0.8621	0.8965	0.9838	0.9756	0.9862
Average	0.9199	0.9564	0.9625	0.9571	0.9835
Inference time (ms)	36.70	129.47	35.83	45.65	30.50
Training time (s)	320	240	153	356	135

**Table 4:** The performance results of different gesture recognition algorithms.

	Accuracy	Precision	Recall	F1 Score	Inference Time (ms)	Training Time (s)
SqueezeNet-BiLSTM	98.94%	97.34%	98.21%	97.21%	85.4	638
CNN-LSTM	94.59%	94.72%	94.59%	94.69%	235.6	678
BiLSTM	92.34%	94.70%	92.34%	92.34%	73.6	436
CNN-BiLSTM	94.68%	95.06%	93.32%	93.32%	386.8	896
SqueezeNet-LSTM	95.97%	96.01%	97.08%	95.97%	65.3	563

**Table 5:** Comparison of the recognition accuracy of SqueezeNet-BiLSTM on new users’ data.

	UserA	UserB	UserC	UserD	Remaining Data
Accuracy	66.1%	63.6%	63.0%	61.4%	96.57%
